# The Synthesis of Glycerol Carbonate from Glycerol and Carbon Dioxide over Supported CuO-Based Nanoparticle Catalyst

**DOI:** 10.3390/molecules28104164

**Published:** 2023-05-18

**Authors:** Jassim Mohamed Hamed Al-Kurdhani, Huajun Wang

**Affiliations:** 1 Hubei Key Laboratory of Material Chemistry & Service Failure, School of Chemistry and Chemical Engineering, Huazhong University of Science and Technology, Wuhan 430074, China; jas_khay@yahoo.com; 2 Key Laboratory for Material Chemistry for Energy Conversion and Storage, Ministry of Education, Huazhong University of Science and Technology, Wuhan 430074, China

**Keywords:** glycerol, glycerol carbonate, carbon dioxide, 2-cyanopyridine, CuO/Al_2_O_3_

## Abstract

A series of supported CuO-based nanoparticle catalysts were prepared by the impregnation method and used for the synthesis of glycerol carbonate from glycerol and CO_2_ in the presence of 2-cyanopyridine as a dehydrant and DMF as a solvent. The effects of supports (activated alumina, silicon dioxide, graphene oxide, graphene, and activated carbon), CuO loading amount, calcination temperature, and reaction parameters on the catalytic activity of the catalyst were investigated in detail. XRD, FTIR, SEM, BET, and CO_2_-TPD were used for the characterization of the prepared catalysts. It is found that CuO/Al_2_O_3_ shows a higher catalytic activity, which depends on the CuO loading amount and calcination temperature. The surface area and number of basic sites of the catalyst exhibit a crucial effect on the catalytic activity of CuO/Al_2_O_3_. Furthermore, there is a synergistic effect between the catalyst and 2-cyanopyridine where the former has a higher activation ability for glycerol and the latter acts not only as a dehydrant, but also as a promoter for CO_2_ activation. Recycling experiments reveal that this catalyst can be reused for at least five cycles without any inactivation. Based on the experiment results and FTIR characterization, a possible reaction mechanism for the carbonylation of glycerol and CO_2_ is proposed.

## 1. Introduction

Glycerol carbonate (GC) is a significant cyclic carbonate with excellent properties and broad use. GC is a nontoxic, readily biodegradable, water-soluble, non-flammable (fp 165.9 °C), and viscous liquid, which can be used as a polar high boiling solvent, a surfactant component, and an intermediate for many kinds of polymers. In addition, GC also can be utilized as a component for gas separation membranes [1,2,3,4]. GC can be synthesized from the reaction of the biological glycerol (GL), a by-product of biodiesel, and various substances with carbonyl, such as dimethyl carbonate (DMC), urea, or carbon dioxide. As the by-product of biodiesel manufacture, the biological GL is produced in huge amounts, for the production of biodiesel is increased rapidly year by year [5,6,7]. Therefore, the synthesis of high value-added GC from the surplus and cheap GL has attracted more and more attention, and is one of the main topics of biomass valorization [8,9,10]. Among the synthesis methods of GC, the reactions of GL with DMC or urea suffer some drawbacks, such as an expensive reactant for the DMC route [11,12,13] or generating an environmentally harmful by-product (NH_3_) for the urea route [14,15,16,17]. Compared with the above methods, the synthesis of GC by the carbonylation of GL with CO_2_ is more interesting and its atom utilization is as high as 87%. Moreover, this reaction is regarded as a green process in which two wastes, GL, a by-product of biodiesel, and CO_2_, a primary greenhouse gas, are converted into a value-added chemical, GC [18,19,20,21,22,23,24,25].

Up to now, a series of homogeneous and heterogeneous catalysts have been developed and used for the reaction of GL with CO_2_. Unfortunately, however, the GL conversion and GC yield are still far from satisfactory because this reaction is severely limited by thermodynamics [20,26,27,28,29]. Dibenedetto et al. used CeO_2_/Al_2_O_3_ or CeO_2_/Nb_2_O_5_ as a catalyst for the reaction of GL with CO_2_ in the presence of tetraglycol dimethyl ether and found that the GL conversion only reached up to 2.5% under a pressure of 5.0 MPa and reaction time of 15 h [28]. In order to break the thermodynamic limit to increase the GL conversion, the dehydrants were used in the reaction to pull out of water produced as a by-product during the reaction to shift the chemical equilibrium to the product side. George et al. used the 13X zeolite as a dehydrant for the reaction of GL and CO_2_ with ^n^Bu_2_SnO as a catalyst and obtained a GC yield of 35% (13.8 MPa, 393 K, 4 h) [21]. The acetonitrile also was used as a dehydrant for the reaction of GL and CO_2_ by using Cu/La_2_O_3_ [19,30], Zn/Al/La/M(M = Li, Mg, Zr) [31], or La_2_O_2_CO_3_-ZnO as a catalyst [32,33], and the GC yield reached up to 15.2%, 18.7%, or 14.3%, respectively. Recently, He et al. used 2-cyanopyridine as a dehydrant and CeO_2_ as a catalyst for the reaction of GL and CO_2_. Although a higher GC yield of 78.9% was obtained in this process, the catalyst amount was too high (187 wt% based on GL weight) for the industrial production of GC [18]. These results indicate that the GL conversion and GC yield increase assuredly due to the introduction of the dehydrant for the reaction of GL and CO_2_ [34]. Compared with acetonitrile and 13X zeolite, 2-cyanopyridine is more suitable for the reaction of GL with CO_2_ because a higher GC selectivity and a lower side reaction would be obtained; moreover, Zhao et al. found that 2-cyanopridine not only acts as the dehydrant, but also activates the carbonyl bond of CO_2_ [35]. However, in spite of this progress, the GC yield is still not high enough for the industrial production, and it is also urgently requisite to develop new more effective catalyst for the synthesis of GC from GL and CO_2_.

On the other hand, there are two types of reactors for the conversion of GL, such as the batch stirred kettle reactor and the continuous flow fixed bed reactor. The batch reactor has flexible operation, high pressure resistance, and a long reaction time, which is more suitable for the experiment study in the laboratory [18,35]. Compared with the batch reactor, the continuous flow fixed bed reactor has higher efficiency, lower cost, ease of construction and operation, lower mass transfer resistance, and is more suitable for industrial production [36,37,38,39,40]. Leão et al. used lipase B from *Candida antarctica* immobilized on Accurel MP1000 as catalyst for the synthesis of GC from vegetable oil and DMC by a three-step process of triacylglycerol hydrolysis, biodiesel synthesis, and transesterification of the remaining GL with DMC in a continuous flow fixed bed reactor [36]. They found that the oil conversion of 99% and GC selectivity of 99% can be obtained under a vegetable oil/DMC molar ratio of 1:10, residence time of 176 min, and reaction temperature of 60 °C. Kostyniuk et al. studied the gas-phase conversion of GL into glycidol over a CsNO_3_/HZSM-5 catalyst in a continuous flow packed-bed reactor under atmospheric pressure, and the yield of glycidol (40.4%) was obtained at a reaction temperature of 350 °C and GHSV of 1250 h^−1^ [37]. In addition, Marinho et al. investigated the GL etherification with ethanol in a fixed bed reactor catalyzed by Amberlyst 15 and the effect of process variables, including pressure, temperature, ethanol/GL molar ratio, and amount of catalyst on the GL conversion, were studied in detail using a four-factor central composite design [38].

**Scheme 1:**
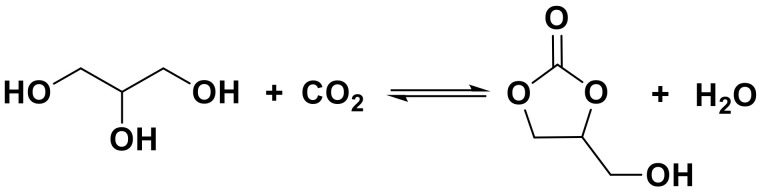
The synthesis of GC from the reaction of GL with CO_2_.

The supported CuO-based nanocatalysts were used in various applications, such as the low-temperature water-gas shift reaction, oxidation of various amounts of SO_2_, oxidation of volatile organic compounds (VOCs), oxidation of methane, epoxidation of alkenes through oxygen activation, catalyst-sorbent suitable for simultaneous SO_2_ and NOx removal from flue gases, etc. [41,42,43,44,45]. However, to the best of our knowledge, there are few reports on the synthesis of GC from GL and CO_2_ by means of using supported CuO-based nanoparticles as the catalyst. In the present work, a series of CuO-based catalysts were prepared and used for the synthesis of GC by the carbonylation of GL and CO_2_ in the presence of 2-cyanopyridine as a dehydrant and DMF as a solvent (Scheme 1). XRD, FTIR, SEM, BET method, and CO_2_-TPD were used to scrutinize the physicochemical properties of the prepared catalysts. The effects of supports (activated alumina, silicon dioxide, graphene oxide, graphene, and activated carbon), CuO loading amount, calcination temperature, and reaction parameters (CO_2_ pressure, reaction temperature, time, and catalyst amount) on the catalytic activity of the catalyst were investigated in detail. Finally, based on the experiment results and FTIR characterization, a possible reaction mechanism for the carbonylation of GL and CO_2_ was proposed.

## 2. Result and Discussion

### 2.1. Effect of Supports

The catalytic activities of supported CuO-based catalysts with different supports, such as graphene oxide (GO), activated carbon (AC), SiO_2_, graphene (GE), and Al_2_O_3_ for the synthesis of GC from GL and CO_2_, are given in Table 1. It is found that among these catalysts, CuO/AC (30%, 500) shows the lowest catalytic activity with a GL conversion of 21.4% and GC yield of 8.9%. In contrast, the CuO/Al_2_O_3_ (30%, 500) gives the highest catalytic activity, and GL conversion and GC yield reach up to 37.8% and 15.0%, respectively, over CuO/Al_2_O_3_ (30%, 500). Although CuO/GO (30%, 500), CuO/GE (30%, 500), and CuO/SiO_2_ (30%, 500) show higher GL conversion than CuO/Al_2_O_3_ (30%, 500), the CuO/Al_2_O_3_ catalyst possesses a higher GC yield and GC selectivity. Therefore, the CuO/Al_2_O_3_ catalyst with activated Al_2_O_3_ as the support is suitable for the synthesis of GC from the reaction of GL with CO_2_. In the next section, the catalytic activities of CuO/Al_2_O_3_ with different CuO loading amounts and calcination temperatures were investigated.

### 2.2. Effect of CuO Loading Amount and Calcination Temperature

The CuO/Al_2_O_3_ catalysts with different CuO loading amounts were also prepared by the impregnation method and then used for the synthesis of GC from GL and CO_2_. It is found that the CuO loading amount has a remarkable effect on the catalytic activity of the CuO/Al_2_O_3_ catalyst (Table 2). As the CuO loading amount is increased from 5 wt% to 30 wt%, the GL conversion and GC yield increase from 14.6% and 2.8% to 37.8% and 15.0%, respectively. When the CuO loading amount is further increased from 30% to 40%, though the GL conversion increases from 37.8% to 51.6%, the GC yield increases slightly from 15.0% to 15.3% and the GC selectivity decreases from 39.8% to 29.7%. It indicates that a CuO loading amount of 30% should be suitable for GC synthesis from the reaction of GL and CO_2_. Meanwhile, the number in brackets in the third column in Table 1 and Table 2 is the actual CuO content measured by ICP-OES. It is observed that the actual CuO content is close to the initial preparation content.

Table 2 also demonstrates the effect of calcination temperature on the catalytic activity of the CuO/Al_2_O_3_ catalyst. It is found that with the increase of the calcination temperature from 400 °C to 800 °C, the GL conversion decreases from 58.0% to 33.5% while the GC yield firstly increases from 15.1% to 17.5% and then decreases to 14.2%. Among these catalysts, CuO/Al_2_O_3_ (30%, 700) possesses the highest GC yield of 17.5% and GC selectivity of 42.4%.

### 2.3. Characterization

#### 2.3.1. XRD

**Figure 1:**
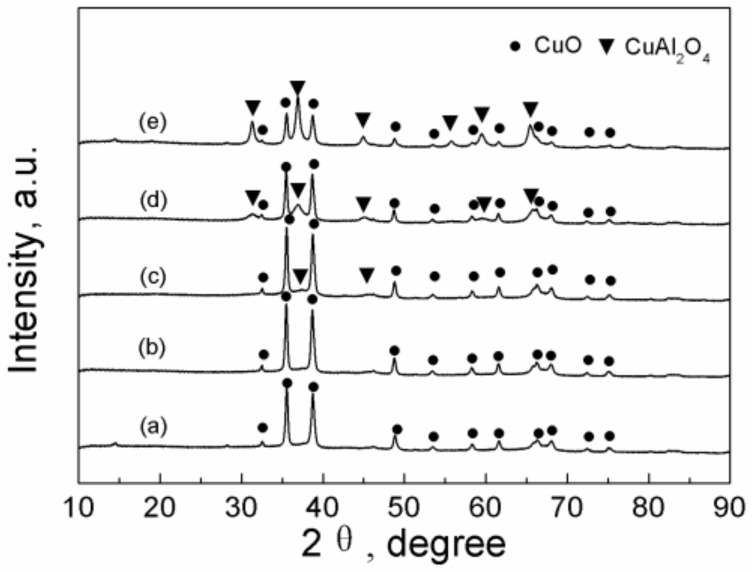
X-ray diffraction patterns of CuO/Al_2_O_3_ nanoparticle catalysts calcined at different temperatures: (**a**) CuO/Al_2_O_3_ (30%, 400), (**b**) CuO/Al_2_O_3_ (30%, 500), (**c**) CuO/Al_2_O_3_ (30%, 600), (**d**) CuO/Al_2_O_3_ (30%, 700), (**e**) CuO/Al_2_O_3_ (30%, 800).

Figure 1 shows the XRD patterns of CuO/Al_2_O_3_ nanoparticle catalysts calcined at different temperatures. It is found that when the calcination temperature is less than 700 °C, the main phase of the samples of CuO/Al_2_O_3_ (30%, 400~600) is monoclinic CuO (at 2θ = 32.5°, 35.5°, 38.7°, 46.2°, 48.7°, 53.3°, 58.3°, 61.5°, 66.2°, 72.3°, 75.2°). Meanwhile, the diffraction intensity of the CuO phase slightly increases with the calcination temperature by comparing the peak at 35.5°. It means that the degree of crystallinity of the sample increases slightly with the calcination temperature. In addition, at the calcination temperature of 600 °C, a new phase, cubic CuAl_2_O_4_ (at 2θ = 31.4°, 36.9°, 44.85°, 55.7°, 59.4°, 65.4°), is observed, which must be formed by the interaction of Cu(NO_3_)_2_ with supporter Al_2_O_3_, indicating that Cu^2+^ is successfully loaded onto the Al_2_O_3_ skeleton [46,47]. As the calcination temperature further increases from 600 °C to 800 °C, the diffraction intensity of the CuO phase becomes weaker. On the contrary, the diffraction intensity of the CuAl_2_O_4_ phase becomes stronger, implying the fact that the content of the CuO phase decreases while the content of the CuAl_2_O_4_ phase increases with the rise in calcination temperature (Figure 1c–e). It indicates that a higher calcination temperature is beneficial for the interaction of Cu(NO_3_)_2_ with supporter Al_2_O_3_ to produce more CuAl_2_O_4_ species. For all samples, no peak related to the Al_2_O_3_ phase was found. It is expected that the calcined Al_2_O_3_ may be presented in the form of η-alumina with low crystallinity.

The mean grain size of CuO in the CuO/Al_2_O_3_ (30%, 400~800) catalysts is calculated by the Scherrer equation based on the reflection peak of CuO at 35.5° and the results are 25.3 nm, 26.6 nm, 26.9 nm, 29.6 nm, and 26.0 nm for CuO/Al_2_O_3_ (30%, 400~800), respectively [47]. It can be seen that the mean grain size of CuO increases with the calcination temperature (except the sample at 800 °C), indicating that the higher temperature is favorable for the growth of the CuO particles. However, the CuO/Al_2_O_3_ (30%, 800) has a smaller CuO grain size of 26.0 nm, and the reason may be that the generation of the CuAl_2_O_4_ phase restrains the growth of the CuO particle.

#### 2.3.2. FT-IR

**Figure 2:**
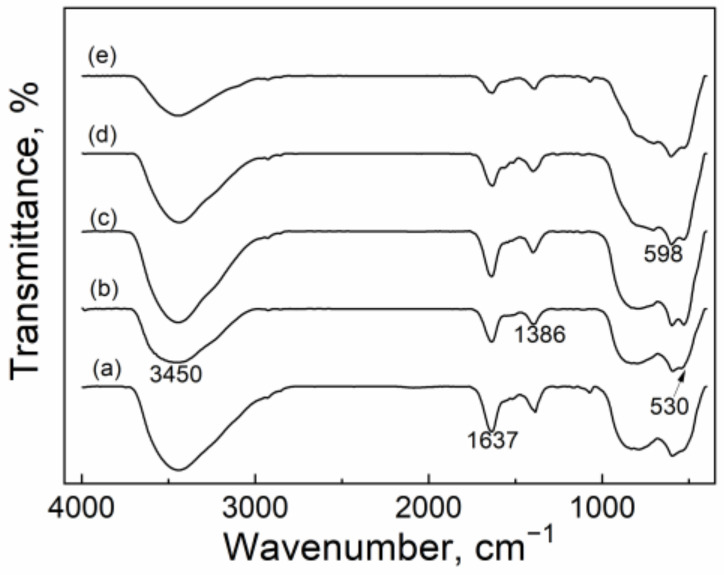
FT-IR spectra of CuO/Al_2_O_3_ nanoparticle catalysts calcined at different temperatures: (**a**) CuO/Al_2_O_3_ (30%, 400), (**b**) CuO/Al_2_O_3_ (30%, 500), (**c**) CuO/Al_2_O_3_ (30%, 600), (**d**) CuO/Al_2_O_3_ (30%, 700), (**e**) CuO/Al_2_O_3_ (30%, 800).

Figure 2 shows the FT-IR spectra of the CuO/Al_2_O_3_ nanoparticles. For all the fresh CuO/Al_2_O_3_ catalysts, the weak peaks at 1637 cm^−1^ and 1386 cm^−1^ and the wide band at 3000~3700 cm^−1^ are assigned to the bending and stretching vibration of -OH of the adsorbed water molecule [48]. The intensities of these peaks decrease gradually with the increase of the calcination temperature, indicating that the water content in the nanoparticle decreases with the rise of the calcination temperature. The peaks at 530 cm^−1^ and 598 cm^−1^ are attributable to the stretching vibration of Cu-O [49]. The FT-IR result suggests that the formation of the CuO compound is obtained after the calcination process, which is consistent with XRD results.

#### 2.3.3. SEM

**Figure 3:**
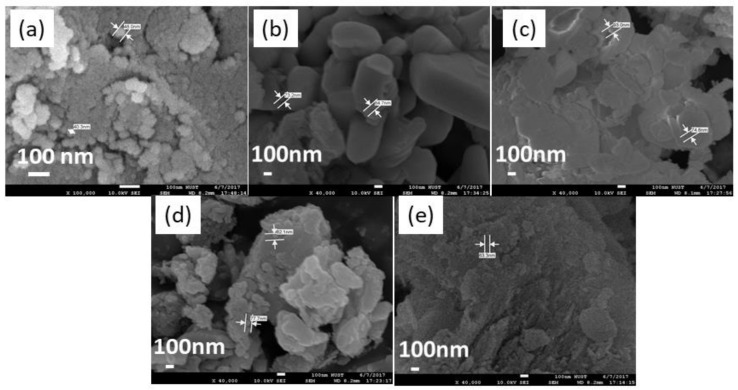
SEM images of CuO/Al_2_O_3_ nanoparticle catalysts calcined at different temperatures: (**a**) CuO/Al_2_O_3_ (30%, 400), (**b**) CuO/Al_2_O_3_ (30%, 500), (**c**) CuO/Al_2_O_3_ (30%, 600), (**d**) CuO/Al_2_O_3_ (30%, 700), (**e**) CuO/Al_2_O_3_ (30%, 800).

Figure 3 shows the SEM images of the CuO/Al_2_O_3_ nanoparticle catalysts with different temperatures. It is found that the calcination temperature has a remarkable effect on the morphology and size of the nanoparticle. The CuO/Al_2_O_3_ (30%, 400) nanoparticle is spherical, and its diameter is about 50 nm. In this sample, the edge of the nanoparticle is unsmooth, meaning that some particles may be secondary particles. For the CuO/Al_2_O_3_ (30%, 500) sample, the particle displays polyhedron or sheet structure with a large diameter, which should be formed by the agglomeration of mini crystals. For the CuO/Al_2_O_3_ (30%, 600), the particles are seriously agglomerated to form a blocky structure, while for the CuO/Al_2_O_3_ (30%, 700) sample, the nanoparticles are agglomerated to form a large nutty structure. In addition, for the CuO/Al_2_O_3_ (30%, 800) sample, the nanoparticles are agglomerated to form a spongy structure. These results indicate that at higher calcination temperatures, the thermal diffusion behavior of the catalyst components takes place to form particles with various shapes, which may have an obvious effect on the catalytic activity of the nanoparticle by affecting the adsorption, desorption, and surface reaction process of the reaction components. For these samples, compared with the nanoparticles with blocky and spongy structures, the samples with a granular structure may be more favorable to the synthesis of GC, for the CuO/Al_2_O_3_ (30%, 400, 500, 700) shows higher GC yields than CuO/Al_2_O_3_ (30%, 600) and CuO/Al_2_O_3_ (30%, 800) (see Table 2).

#### 2.3.4. BET

**Figure 4:**
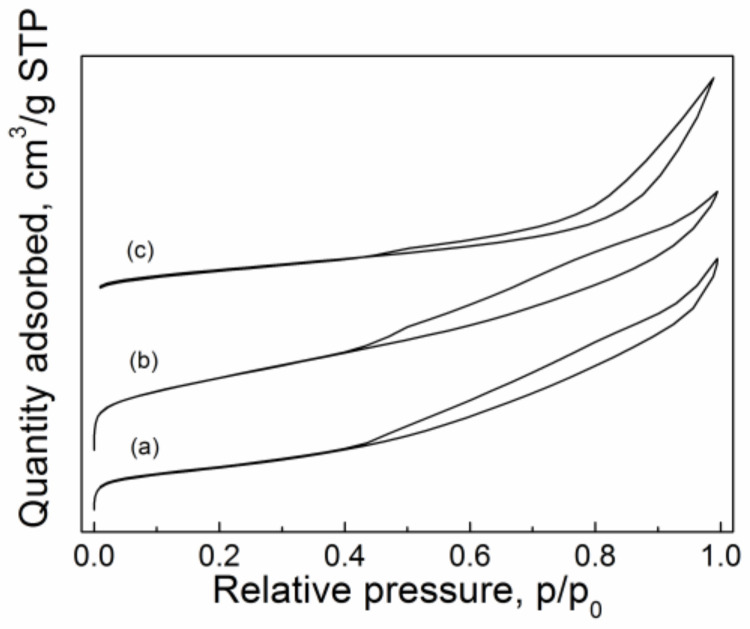
N_2_ adsorption-desorption isotherms of CuO/Al_2_O_3_ nanoparticle catalysts calcined at different temperatures: (**a**) CuO/Al_2_O_3_ (30%, 600), (**b**) CuO/Al_2_O_3_ (30%, 700), and (**c**) CuO/Al_2_O_3_ (30%, 800).

The N_2_ adsorption-desorption isotherms of CuO/Al_2_O_3_ (30%, 600~800) are shown in Figure 4. The related BET-specific surface areas, total pore volumes, and average pore diameters are listed in Table 3. All the catalysts exhibited the type IV isotherms with the type III hysteresis loops in terms of IUPAC classification [29]. The result indicates that the prepared CuO/Al_2_O_3_ belongs to the mesoporous nanoparticle. With the increase of the calcination temperature from 600 °C to 700 °C, although the mean grain size of CuO increases from 26.9 nm to 29.6 nm (see XRD results), the specific surface area remarkably increases from 111.3 m^2^/g to 170.5 m^2^/g while the total pore volume slightly increases from 0.269 cm^3^/g to 0.277 cm^3^/g (in Table 3). The increase in the specific surface area may be related to the morphology of the sample nanoparticle. The loosened nutty structure constituted by the granules for CuO/Al_2_O_3_ (30%, 700) may be more favorable to generate a large surface area than the pyknotic blocky structure for CuO (30%, 600) (Figure 3). When the calcination temperature increases further to 800 °C, the surface area and the total pore volume of CuO/Al_2_O_3_ (30%, 800) decrease to 92.7 m^2^/g and 0.240 cm^3^/g, respectively. Among these catalysts, the CuO/Al_2_O_3_ (30%, 700) shows the largest surface area and total pore volume, which may cause the exposure of more active sites and lead to a higher catalytic activity. Table 2 shows that among these catalysts, CuO/Al_2_O_3_ (30%, 700) has the highest GC yield, meaning that the catalytic activity may correlate to the surface area and total pore volume. However, although the surface area and the total pore volume of CuO/Al_2_O_3_ (30%, 800) are lower than that of CuO/Al_2_O_3_ (30%, 600), the GC yield over CuO/Al_2_O_3_ (30%, 800) is slightly higher than that over CuO/Al_2_O_3_ (30%, 600) (see Table 2), which indicates that the catalytic activity does not only correlate to the surface area of the catalyst.

#### 2.3.5. CO_2_-TPD

**Figure 5:**
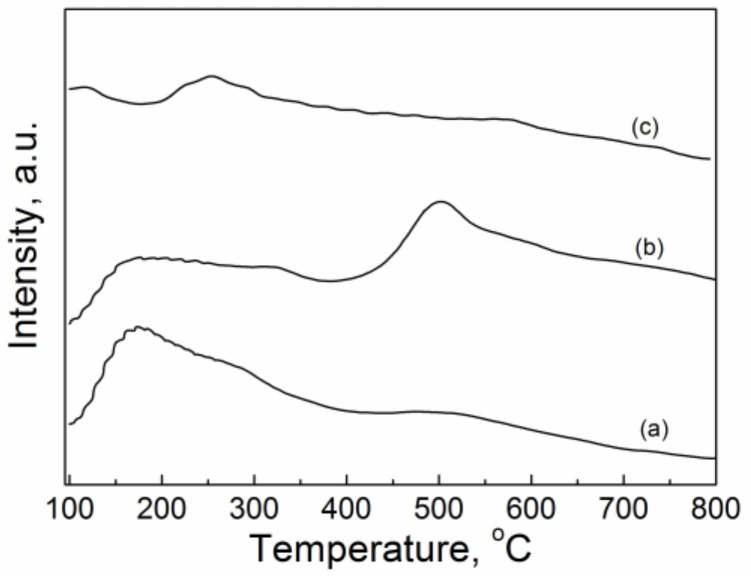
CO_2_-TPD profiles of CuO/Al_2_O_3_ nanoparticle catalysts calcined at different temperatures: (**a**) CuO/Al_2_O_3_ (30%, 600), (**b**) CuO/Al_2_O_3_ (30%, 700), (**c**) CuO/Al_2_O_3_ (30%, 800).

Figure 5 shows the CO_2_-TPD profiles of the CuO/Al_2_O_3_ (30%, 600~800) catalysts. The CO_2_ desorption peaks can be roughly divided into three regions: the weak (100~200 °C), moderate (200~400 °C), and strong (>400 °C) basic sites. The basic sites’ amounts can be estimated by integrating the peak area, and the results are listed in Table 3. It can be found that with increase of calcination temperature from 600 °C to 700 °C, the total basic sites amount and the strong basic sites amount increased from 875 umol/g and 337 umol/g to 1082 umol/g and 704 umol/g, respectively, while the moderate basic sites amount decreased from 367 umol/g to 185 umol/g. When the calcination temperature further increased to 800 °C, the total basic sites amount, the strong basic sites amount, and the moderate sites amount decreased to 329 umol/g, 234 umol/g, and 90 umol/g, respectively. For these samples, the more surface area a catalyst possesses, the more basic sites it has, and vice versa. Among these catalysts, CuO/Al_2_O_3_ (30%, 700) possesses the highest surface area, so it has the highest amount of total basic sites. On the contrary, CuO/Al_2_O_3_ (30%, 800) has the least surface area, and thereby it possesses the least amount of total basic sites. In addition, the CuO/Al_2_O_3_ (30%, 700) has a higher amount of strong basic sites and lesser amount of moderate basic sites than CuO/Al_2_O_3_ (30%, 600), which may be ascribed to that the former possesses more CuAl_2_O_4_ species and lesser CuO phase (see Figure 1d,e). It means that the CuAl_2_O_4_ phase may have a higher strong basic site and the CuO phase may contain a higher amount of moderate basic sites. By the same token, though the CuO/Al_2_O_3_ (30%, 800) possesses the least amount of total basic sites, its ratio of the amount of strong basic sites is 0.71, which is the highest among these samples, for the content of the CuAl_2_O_4_ phase is highest (see Figure 1c–e).

The previous research results indicated that the basic sites amount on the catalyst surface is very important for the activation of CO_2_ in the synthesis of dimethyl carbonate from methanol and CO_2_ [50]. Therefore, the CuO/Al_2_O_3_ (30%, 700) exhibits the higher GC yield, which may be attributed to a higher total basic sites amount. Furthermore, although the total basic sites amount of CuO/Al_2_O_3_ (30%, 800) is smaller, this catalyst shows higher GC yield, which may be ascribed to the higher ratio of strong basic sites amount. The CuAl_2_O_4_ phase with more strong basic sites may have a strong ability to extract a hydrogen atom from the hydroxyl (-OH) of GL to activate GL [51]. In addition, Wei et al. found that the moderate basic sites are beneficial to active CO_2_ [31]; thereby, the catalysts with more CuO phase with a more moderate basic sites amount, such as CuO/Al_2_O_3_ (30%, 400) and CuO/Al_2_O_3_ (30%, 500) (see Figure 1), also exhibit a higher GC yield. The above results indicate that a large surface area and higher amount of strong basic sites and moderate basic sites are crucial for the reaction of GL with CO_2_.

### 2.4. Effect of Reaction Conditions

#### 2.4.1. Effect of Reaction Temperature

**Figure 6:**
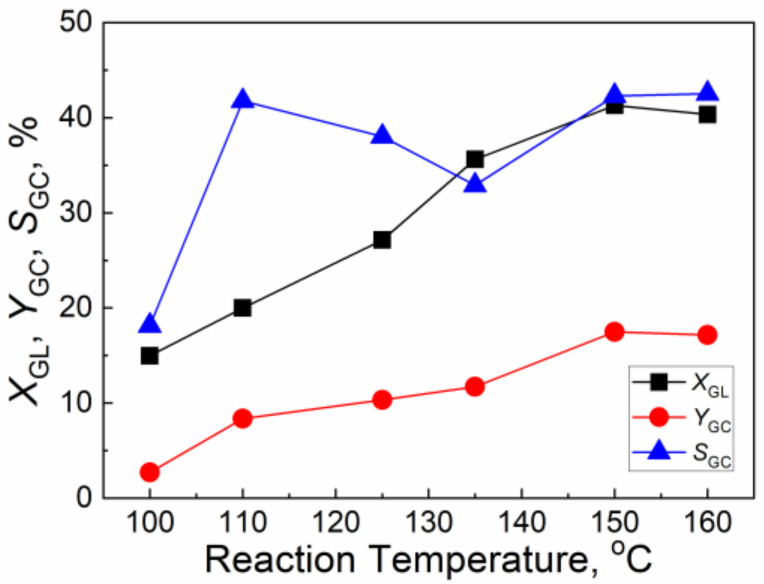
The effect of reaction temperature on the catalytic activity of CuO/Al_2_O_3_(30%, 700) for the reaction of GL with CO_2_ (Reaction condition: GL: 2.30 g; Cat: 1.0 g; CO_2_ initial pressure: 4.0 MPa; reaction time: 5 h; 2-cyanopyridine: 6.32 g; DMF: 19.0 g).

Figure 6 shows the effect of reaction temperature on the catalytic activity of CuO/Al_2_O_3_ (30%, 700) for the reaction of GL with CO_2_. With the increase of the reaction temperature from 100 °C to 150 °C, the GL conversion and GC yield increase from 15.0% and 2.7% to 41.3% and 17.5%, respectively, while the GC selectivity increases from 18.1% to 42.3%. With the further increase of the reaction temperature to 160 °C, the yield and selectivity of GC almost keep constant. Thus, the best reaction temperature is 150 °C.

#### 2.4.2. Effect of CO_2_ Initial Pressure

**Figure 7:**
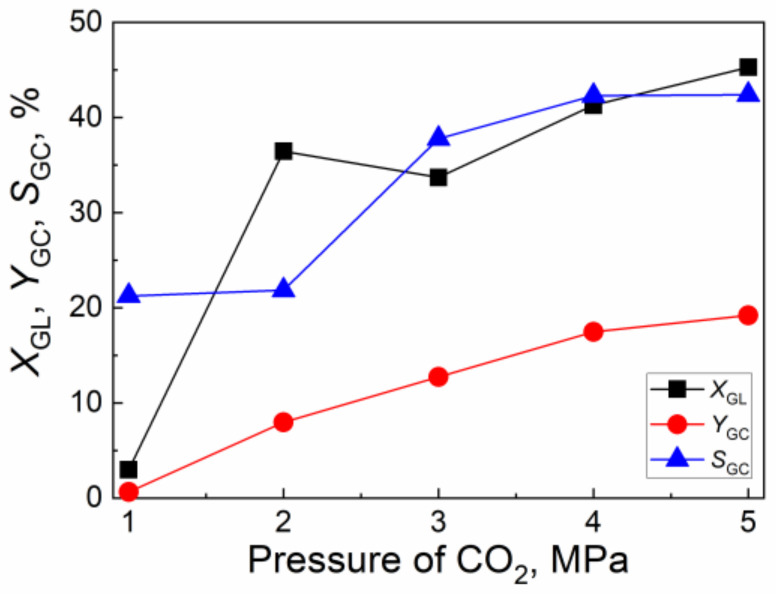
The effect of CO_2_ initial pressure on the catalytic activity of CuO/Al_2_O_3_ (30%, 700) for the reaction of GL with CO_2_ (Reaction condition: GL: 2.30 g; Cat: 1.0 g; reaction temperature: 150 °C; reaction time: 5 h; 2-cyanopyridine: 6.32 g; DMF: 19.0 g).

Figure 7 illustrates the effect of CO_2_ initial pressure on the catalytic activity of the CuO/Al_2_O_3_ (30%, 700) catalyst for the reaction of GL with CO_2_. As CO_2_ initial pressure increases from 1.0 MPa to 4.0 MPa, the GL conversion increases from 3.0% to 41.3% while the GC yield and GC selectivity increase from 0.6% and 21.3% to 17.5% and 42.3%, respectively. With the further increase of CO_2_ pressure to 5.0 MPa, the GL conversion, GC yield, and GC selectivity slightly increase. Thus, the suitable CO_2_ initial pressure is 4.0~5.0 MPa.

#### 2.4.3. Effect of Reaction Time

**Figure 8:**
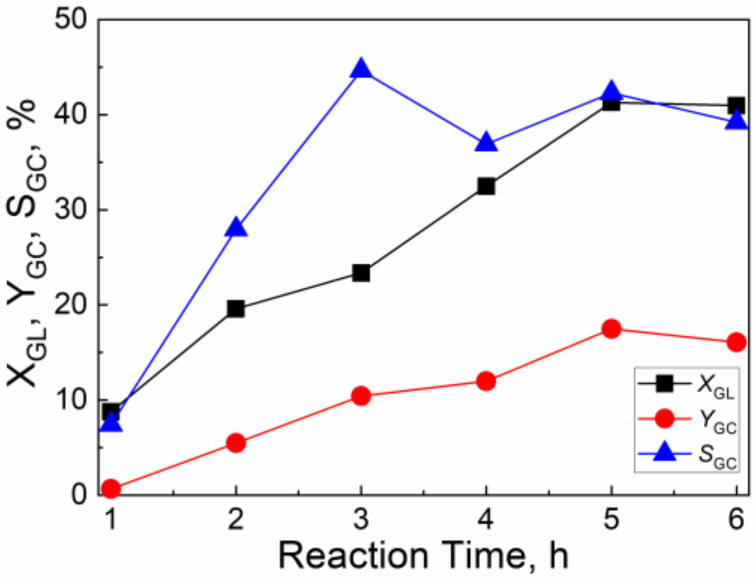
The effect of reaction time on the catalytic activity of CuO/Al_2_O_3_ (30%, 700) for the reaction of GL with CO_2_ (Reaction condition: GL: 2.30 g; Cat: 1.0 g; CO_2_ initial pressure: 4.0 MPa; reaction temperature: 150 °C; 2-cyanopyridine: 6.32 g; DMF: 19.0 g).

Figure 8 shows the effect of reaction time on the catalytic activity of CuO/Al_2_O_3_ (30%, 700) for the reaction of GL with CO_2_. As the reaction time increases from 1 h to 5 h, the GL conversion increases from 8.8% to 41.3%, while the GC yield and GC selectivity continually increase from 0.7% and 7.4% to 17.5% and 42.3%, respectively. With the further increase of reaction time to 6 h, the yield and selectivity of GC are slightly decreased. Thus, the suitable reaction time is 5 h.

#### 2.4.4. Effect of Weight of Catalyst

**Figure 9:**
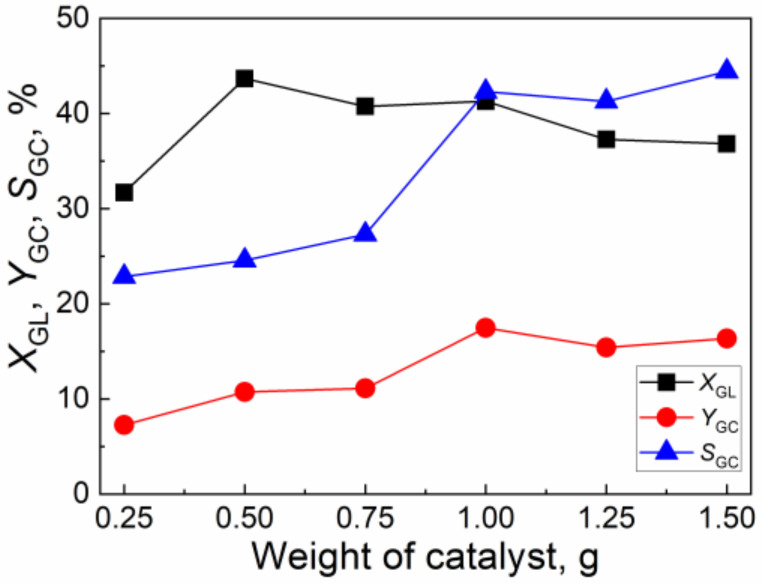
The effect of the weight of the catalyst on the catalytic activity of CuO/Al_2_O_3_ (30%, 700) for the reaction of GL with CO_2_ (Reaction condition: GL: 2.30 g; CO_2_ initial pressure: 4.0 MPa; reaction temperature: 150 °C; reaction time: 5 h; 2-cyanopyridine: 6.32 g; DMF: 19.0 g).

The effect of the weight of the catalyst on the catalytic activity of CuO/Al_2_O_3_ (30%, 700) is illustrated in Figure 9. As the weight of the catalyst increases from 0.25 g to 1.0 g, the GL conversion decreases from 31.7% to 41.3%, while GLC yield and GLC selectivity increase from 7.2% and 22.9% to 17.5% and 42.3%, respectively. With the further increase of the weight of the catalyst, the GL conversion, GLC yield, and GLC selectivity decrease slightly. Thus, the suitable weight of the catalyst is 1.0 g. In order to validate the reliability and reproducibility of the results, several experiments were repeated three times under the same reaction conditions. The relative deviation for GL conversion, GC yield, and GC selectivity are less than 1%, 2.5%, and 2.8%, respectively. In addition, the relative error of the material masses before and after the reaction is found to be within 2.9%, indicating the reliability of the results.

### 2.5. Stability of CuO/Al_2_O_3_ (30%, 700) Catalyst

**Figure 10:**
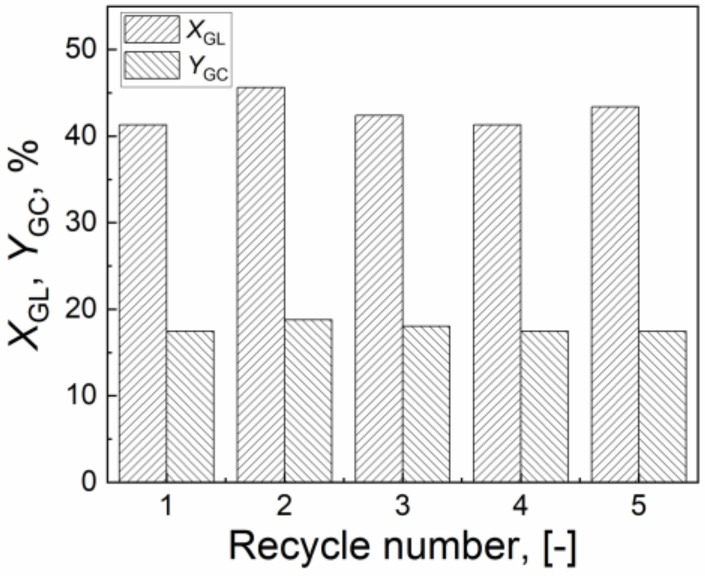
The stability of the CuO/Al_2_O_3_ (30%, 700) catalyst on the reaction of GL with CO_2_ (Reaction condition: GL: 2.30 g; Cat: 1.0 g; CO_2_ initial pressure: 4.0 MPa; reaction temperature: 150 °C; reaction time: 5 h; 2-cyanopyridine: 6.32 g; DMF: 19.0 g).

The recycling experiments were also carried out to scrutinize the stability of the CuO/Al_2_O_3_ (30%, 700) catalyst. After the reaction, the catalyst was separated by centrifugation, washed with methanol three times, and then reused for the next run under the same condition. As shown in Figure 10, at the fifth recycle, the GL conversion and GC yield still can reach 43.4% and 17.4%, respectively, over the recovered CuO/Al_2_O_3_ (30%, 700) catalyst. These results indicate that CuO/Al_2_O_3_ (30%, 700) has strong stability in the activity during the reaction process.

### 2.6. Proposed Reaction Mechanism

**Figure 11:**
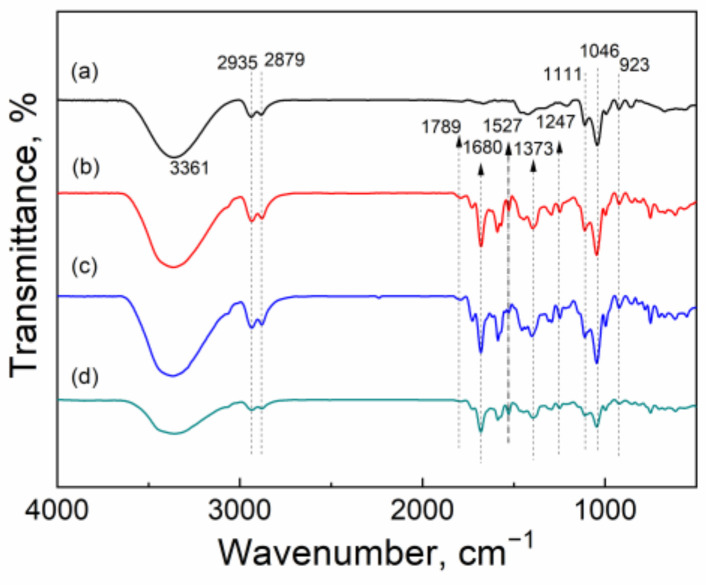
FT-IR spectra of the reaction mixture with different reaction times: (**a**) 0 min; (**b**) 60 min; (**c**) 120 min; (**d**) 180 min.

**Scheme 2:**
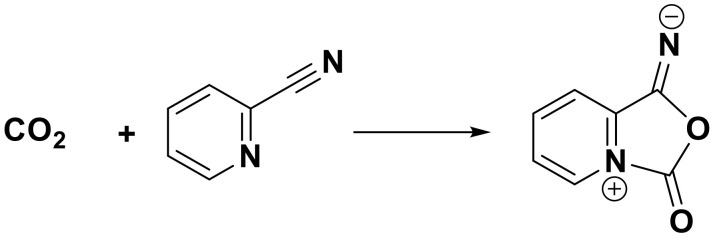
The interaction of CO_2_ with 2-cyanopyridine to form an intermediate.

In order to clearly understand the reaction mechanism of carbonylation of GL with CO_2_ over CuO/Al_2_O_3_ in the presence of 2-cyanopyridine as a dehydrant with DMF as a solvent, the FT-IR spectra were used to characterize the reaction mixture with different reaction times. As shown in Figure 11a, the peaks at 3361 cm^−1^, 2935 cm^−1^, 2879 cm^−1^, and 923 cm^−1^ are attributed to the vibration of the -OH of GL molecules [52], and the bands at 1046 cm^−1^ and 1111 cm^−1^ to the C-O stretching of GL [31], whose strengths decrease with the increase of reaction time (Figure 11b–d), indicating that the amount of GL gradually decreases in the reaction mixture due to the reaction consumption. Furthermore, some new bands centered at 1789 cm^−1^, 1680 cm^−1^, 1527 cm^−1^, 1373 cm^−1^, and 1247 cm^−1^ appear for mixture at 60 min, 120 min, and 180 min, compared with the sample at 0 min. The peak at 1789 cm^−1^ is ascribed to the stretching vibration of C=O of GC [52]. The peak at 1373 cm^−1^ is assigned to the C-N bond in the ring and the peak at 1527 cm^−1^ is attributed to the C=N bond (N in C≡N). Meanwhile, the band at 1247 cm^−1^ is ascribed to the new C-N bonds formed from the interaction between the cyano group of 2-cyanopyridine and CO_2_, while the band at 1680 cm^−1^ is assigned to the C=O bond [35]. These peaks mean that an intermediate species may be formed by the interaction of CO_2_ with 2-cyanopyridine molecules (Scheme 2), resulting in the activation of CO_2_. These results are in accordance with the reports by Zhao et al., in which a five-membered ring intermediate was also proven to be formed from the reaction of 2-cyanopyridine with CO_2_ for the activation of CO_2_ [35]. Meanwhile, He et al. found that when DMF was mixed with GL and 2-cyanopyridine and then treated with CO_2_, the five-membered ring intermediate was not produced, meaning DMF as a solvent could weaken the interaction between 2-cyanopyridine and CO_2_ and therefore, CO_2_ could not be activated in the mixture of GL + 2-cyanopyridine + DMF [22]. However, the results in the present work indicate that though there is DMF in the mixture, CO_2_ can still be activated by 2-cyanopyride.

**Figure 12:**
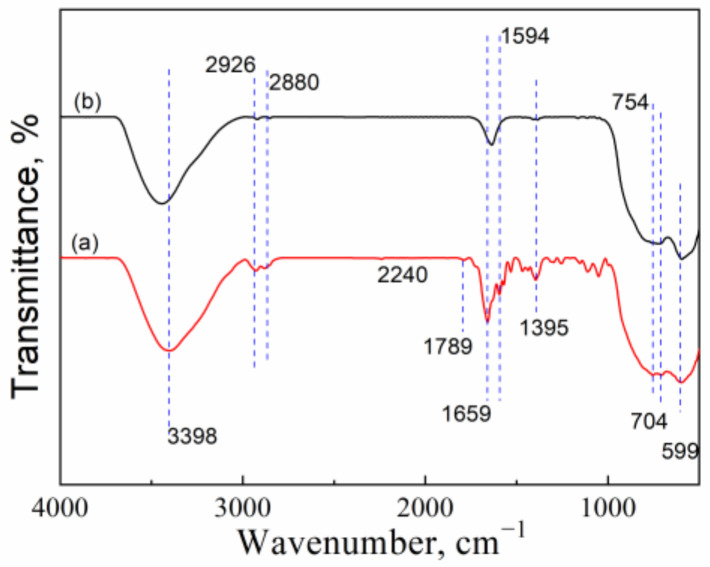
FT-IR spectra of (**a**) recovered CuO/Al_2_O_3_ (30%, 700) and (**b**) fresh CuO/Al_2_O_3_ (30%, 700).

The recovered CuO/Al_2_O_3_ (30%, 700) catalyst was also characterized by FT-IR as well as the fresh catalyst, and the results are illustrated in Figure 12. Compared with the fresh catalyst, the recovered CuO/Al_2_O_3_ (30%, 700) exhibits several new peaks in FT-IR spectra (Figure 12a). The band at 1789 cm^−1^ is attributed to GC, while the peak centered at 754 cm^−1^ is assigned to picolinamide, the product of the hydration reaction of 2-cyanopyridine and water. The bands at 2926 cm^−1^ and 2880 cm^−1^ become large and shift to high frequency, indicating that GL molecules are adsorbed on the active sites of catalyst. The peak at 1594 cm^−1^ may be evidence for the formation of Cu(C_3_O_3_H_6_) from the interaction of GL with the CuO/Al_2_O_3_ (30%, 700) catalyst [23]. Furthermore, the little peak at 2240 cm^−1^ is ascribed to 2-cyanopyridine, while the peak at 1395 cm^−1^ belongs to CO_2_ [22]. These results indicate that GL, 2-cyanopyridine, and CO_2_ can be activated on the CuO/Al_2_O_3_ catalyst. However, the activated CO_2_ molecule may prefer to interact with 2-cyanopyridine to form a more highly active intermediate rather than to react directly with GL [35]. It means that the CuO/Al_2_O_3_ (30%, 700) catalyst can accelerate the interaction of CO_2_ and 2-cyanopyridine, which may be the reason why the active intermediate can be generated in the presence of DMF.

**Scheme 3:**
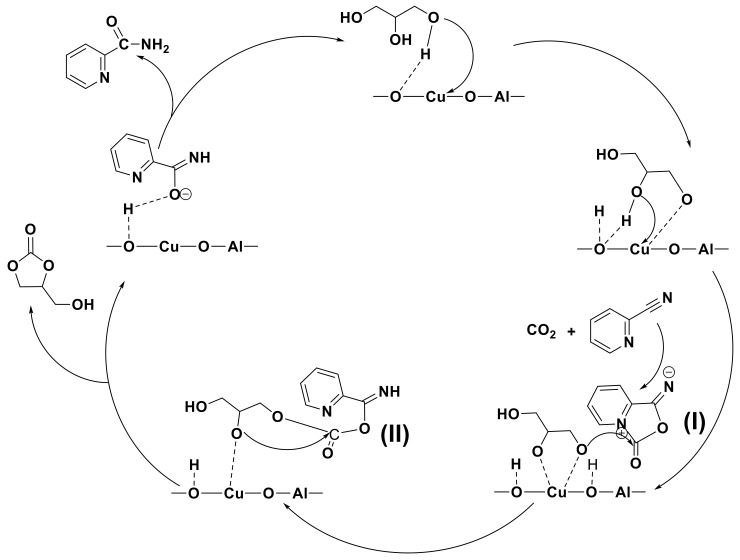
The proposed reaction mechanism for the synthesis of GC from GL and CO_2_ over CuO/Al_2_O_3_(30%, 700).

Based on these experimental and characterization results, a possible reaction route for the synthesis of GC from GL and CO_2_ is proposed and shown in Scheme 3. During the reaction, firstly, the primary O-H bond of GL is activated by adsorption on the strong basic sites of the CuO/Al_2_O_3_ catalyst. Afterwards, the second O-H bond of GL is also activated by an interaction with CuO/Al_2_O_3_ to form the Cu(C_3_O_3_H_6_), while the CO_2_ molecule is activated by interacting with 2-cyanopyridine to form a five-membered ring intermediate I. Subsequently, the activated GL molecule makes a nucleophilic attack to the carbonyl carbon atom of the intermediate I to form intermediate II, and then the oxygen atom of the second O-H bond of GL makes an intramolecular nucleophilic attack on carbonyl carbon to give GC. Meanwhile, 2-cyanopyridine is transformed to 2-picolinamide. It is indicated that during the reaction, 2-cyanopyridine acts not only as a dehydrant, but also as a promoter for CO_2_ activation.

### 2.7. Comparison of the Catalytic Activity between Different Catalysts

The comparison of the catalytic activity of CuO/Al_2_O_3_ (30%, 700) with the other catalysts for GC synthesis from GL and CO_2_ is illustrated in Table 4. Although the different reaction conditions were used for these catalysts, these reaction conditions should be optimized for each catalyst. Thus, the catalytic activity of these catalysts can be compared by using the GC yields and STYs at these optimal conditions. It is observed that due to the presence of the dehydrant, the catalysts, such as CeO_2_, La_2_O_2_CO_3_-ZnO, CHT-Cl, CeO_2_-nanopolyhedra, and CuO/Al_2_O_3_ (30%, 700), show a higher GC yield than 14.0%. On the contrary, the other catalysts, ZnY, ZnO, and ZnWO_4_-ZnO, give a lower GC yield than 9.0%, for the dehydrant was not used during the reaction. It indicates that the dehydrant is very significant for the reaction of GL with CO_2_. Among these catalysts, the activity of CuO/Al_2_O_3_ (30%, 700) is only lower than that of CeO_2_. However, CuO/Al_2_O_3_ (30%, 700) has a remarkably lower catalyst amount than CeO_2_. The higher catalytic activity of CuO/Al_2_O_3_ (30%, 700) is ascribed to the synergistic effect of the catalyst and 2-cyanopyridine, where the former has a higher activation ability for GL and the latter acts as a promoter for CO_2_ activation. The above results suggest that CuO/Al_2_O_3_ (30%, 700) not only has higher catalytic activity and stability, but also has a simple prepared process and cheap cost and thereby, it may be as a good alternative catalyst for the industrial production of GC from GL and CO_2_.

## 3. Materials and Methods

### 3.1. Chemicals

Copper nitrate [(Cu(NO_3_)_2_·3H_2_O] (99% purity), activated alumina (Al_2_O_3_), silicon dioxide (SiO_2_), activated carbon (AC), graphite, GL (99% purity), and methanol (99.5% purity) were bought from Sinopharm Chemical Reagent Co., Ltd., Beijing, China. 2-cyanopyridine (98% purity), tetraethylene glycol (99% purity), and 2-picolinamide (>98% purity) were purchased from Aladdin Industrial Corporation, Shanghai, China. Carbon dioxide (99.9% purity) was supplied by Wuhan Minghui Gas Technology Co., Ltd., Wuhan, China. GC (Tokyo Chemical Industry Co., Ltd., Tokyo, Japan) was of over 90% purity. *N*, *N*-dimethyl formamide (DMF) (99.5% purity) was purchased from Chinasun Specialty Products Co. Ltd., Suzhou, China. All these chemicals were used without further purification.

### 3.2. Supported CuO-Based Nanoparticle Catalyst Preparation

The Al_2_O_3_, SiO_2_, AC, graphene oxide (GO), and graphene (GE) were used as supports to prepare the supported CuO-based nanoparticle catalysts. The CuO/Al_2_O_3_ catalysts were prepared by the impregnation method using an aqueous solution of Cu(NO_3_)_2_·3H_2_O and activated alumina powder. Firstly, the activated alumina powder was dried under a vacuum at 60 °C for 2 h to remove physisorbed water. Then, a certain amount of activated alumina (such as 7 g) was mixed with an aqueous solution of Cu(NO_3_)_2_·3H_2_O (for example, 100 mL and the amount of Cu(NO_3_)_2_·3H_2_O of 9.08 g) in a glass flask. After stirring for 15 min, the mixture was set for 24 h at room temperature and then dried at 100 °C for 1 h to remove water through evaporation. Subsequently, the solid mixture was grinded and sieved using the standard sieve with 100 mesh. The obtained solid powder was calcined at a specified temperature (for example, 700 °C) for 5 h under static air. A heating ramp of 5 °C/min was employed in this step. Finally, the obtained catalyst was used in the reaction of GL and CO_2_. All the prepared catalysts are denoted as CuO/Al_2_O_3_ (n%, m), where n% is the weight percentage of CuO loaded on Al_2_O_3_ and m is the calcination temperature. The CuO/SiO_2_ (30%, 500) catalyst and CuO/AC (30%, 500) were prepared by the same method with CuO/Al_2_O_3_ (30%, 500), using SiO_2_ and AC as supports instead of Al_2_O_3_, respectively.

CuO/GO (30%, 500) was prepared by the procedure shown below. Firstly, the GO was obtained by the modified Hummers method. Briefly, 100 mL of the concentrated H_2_SO_4_ and 10 mL of the concentrated H_3_PO_4_ were poured into a beaker, and then, 2 g of graphite was added. The beaker was put in an ultrasonic cleaner for 1 h at 20 °C and 200 W. Subsequently, 0.75 g of KMnO_4_ was added into the beaker, which was further treated by ultrasonic for another 2 h. Afterwards, the additional 3 g of KMnO_4_ was added and the solution was stirred at 60 °C for 3 h. Then, the mixture was poured into a large beaker with 190 mL of ice water, and then, 7.5 mL of H_2_O_2_ was added to give the graphite oxide. The sample was washed by centrifugation to be neutral and treated by ultrasonic dispersion at 200 W for 1h, and then dried at 60 °C to give GO. The CuO/GO (30%, 500) was prepared by the same procedure as CuO/Al_2_O_3_ (30%, 500), using GO as the support instead of Al_2_O_3_.

CuO/GE (30%, 500) was obtained by the following method: firstly, the GE was fabricated via a thermal exfoliation method. During the process, the dried GO was thermally exfoliated at 300 °C for 3 min in air, and subsequently, the sample was further treated at 900 °C for 3 h in air to give GE. The CuO/GE (30%, 500) was prepared by the same procedure as CuO/Al_2_O_3_ (30%, 500), using GE as the support instead of Al_2_O_3_.

### 3.3. Catalyst Characterization

X-ray diffraction (XRD, X’Pert PRO, PANalytical B.V., Almelo, Netherlands) patterns of the catalysts were measured on a X’Pert PRO using Cu Kα radiation at 30 kV and 15 mA over a 2θ range of 5°~90° with a step size of 0.0167° at a scanning speed of 8 min^−1^. A Bruker VERTEX 70 FT-IR spectrometer (Bruker, Karlsruhe, Germany) was used to obtain the FT-IR spectra of samples using the KBr pellet technique with 2 cm^−1^ resolution over the wavenumber range 4000–400 cm^−1^. The morphology of the particles was observed by the use of a scanning electron microscope (SEM, TESCAN VEGA3, TESCAN, Brno, Czech Republic) with 20.0 kV of an accelerating voltage. Nitrogen adsorption-desorption isotherms were determined by a volumetric adsorption apparatus (Micromeritics ASAP 2420, Micromeritics, Norcross, GA, USA) at 77 K. The surface areas of samples were calculated by using the Brunauer-Emmett-Teller (BET) method. The pore volume was given at p/p0 = 0.99. The pore size distribution was calculated by the Barrett-Joyner-Halenda (BJH) method. An inductively coupled plasma optical emission spectrometer (ICP-OES, Thermo Fisher iCAP 7000, Thermofisher, Germany) was used to measure the Cu content in the acid-dissolved supported CuO-based catalysts, and then CuO content was also obtained.

The basicity studies of the prepared catalysts were conducted with temperature-programmed desorption of CO_2_ as the probe molecule (CO_2_-TPD) using the Huasi DAS-7000 apparatus equipped with a thermal conductivity detector (TCD, Huasi, Changsha, China). The analysis was performed by heating 100 mg of the catalyst sample under a He flow from room temperature to 800 °C for 2 h (10 °C/min, 50 mL/min). Then, the temperature decreased to 90 °C, and a flow of pure CO_2_ (50 mL/min) was subsequently introduced into the reactor during 1 h. After the catalyst was swept with He for 1 h to remove the physisorbed CO_2_ from the catalyst surface, the TPD of CO_2_ was carried out between 90 °C and 900 °C under a He flow (10 °C/min, 30 mL/min), and the detection of the desorbed CO_2_ was performed by an on-line gas chromatograph provided with a TCD.

### 3.4. Reaction Procedure

The tests of the catalytic activities of the supported metal oxide nanoparticle catalysts were carried out in a stainless-steel autoclave reactor with an inner volume of 250 mL, equipped with thermostat, an electric heating jacket, pressure gauge, thermocouple, and agitator. After ascertaining the validity of the autoclave reactor, the typical procedure is as follows: 2.30 g of GL, 1.0 g of catalyst, 19.0 g of DMF, and 6.32 g of 2-cyanopyridine were put into the reactor together, and then, the reactor was sealed and purged with CO_2_ 3 times and pressurized with CO_2_ to 4 MPa. Subsequently, the reactor was heated to the reaction temperature (150 °C) and maintained for a certain reaction time (5 h) under vigorous stirring (600 rpm). After the reaction, the reactor was cooled to room temperature and depressurized. Then, all the product mixture was taken out from the autoclave reactor and the solid catalyst was separated by centrifugation from the liquid mixture. The collected catalyst was washed with methanol three times and then used in the recycle experiment. All of the liquid products were sampled for analysis. All the components were analyzed by the gas chromatograph (Fuli 9790-II, Fuli, Wenling, China) equipped with a flame ionization detector (FID) and a capillary column KB-WAX (30 m long, 0.25 mm i.d.). The internal standard method was used. The temperatures of the injector and the detector were 250 °C and 270 °C, respectively. The temperature of the column was programmed to have a 2-min initial hold at 70 °C, a 15 °C/min ramp from 70 °C to 250 °C, and then a 15 min hold at 250 °C.

The conversion of GL, $X_{\mathrm{GL}}$, the yield of GC, $Y_{\mathrm{GC}}$, and the selectivity to GC, $S_{\mathrm{GC}}$, were calculated according to the following equations:

(1) $$X_{\mathrm{GL}}=\frac{n_{\mathrm{GL}}^{in}-n_{\mathrm{GL}}^{out}}{n_{\mathrm{GL}}^{in}}\times 100\%$$

(2) $$Y_{\mathrm{GC}}=\frac{n_{\mathrm{GC}}^{out}}{n_{\mathrm{GL}}^{in}}\times 100\%$$

(3) $$S_{\mathrm{GC}}=\frac{n_{\mathrm{GC}}^{out}}{n_{\mathrm{GL}}^{in}-n_{\mathrm{GL}}^{out}}\times 100\%$$

where $n_{\mathrm{GL}}^{in}$ is the initial mole number (mol) of GL, while $n_{\mathrm{GL}}^{out}$ and $n_{\mathrm{GC}}^{out}$ are the mole numbers (mol) of GL and GC in the residual reaction mixture after the reaction, respectively.

## 4. Conclusions

To improve the conversion of GL to GC, supported CuO-based nanoparticles were prepared and used as the catalyst in this transformation. CuO/Al_2_O_3_ shows excellent catalytic activity for the synthesis of GC from GL and CO_2_. Both the calcination temperature and CuO loading amount exhibit a significant effect on the catalytic activity of the CuO/Al_2_O_3_ catalyst. A larger surface area and higher number of basic sites are crucial for the reaction of GL with CO_2_. FT-IR characterization clearly shows that during the reaction, 2-cyanopyridine acts not only as a dehydrant, but also as a promoter to activate the chemically inert CO_2_. The GL conversion and GC yield can reach up to 41.3% and 17.5%, respectively, under the CO_2_ pressure of 4.0 MPa at 150 °C for 5 h over the CuO/Al_2_O_3_ (30%, 700) catalyst. This catalyst with a higher catalytic activity, stability, simple prepared process, and cheap cost may be used as an alternative component for the industrial process.

## Figures and Tables

**Table 1:** The catalytic activities of supported CuO-based catalysts with different supports for the synthesis of GC from GL and CO_2_ ^a^.

Catalyst	*T*_cal_ (°C)	*W*_CuO_ (wt%)	*X*_GL_ (%)	*Y*_GC_ (%)	*S*_GC_ (%)
CuO/GO (30%, 500)	500	30 (31.1) ^b^	49.0	14.6	29.7
CuO/AC (30%, 500)	500	30	21.4	8.9	41.4
CuO/SiO_2_ (30%, 500)	500	30	38.2	10.1	26.5
CuO/GE (30%, 500)	500	30	49.0	13.4	27.3
CuO/Al_2_O_3_ (30%, 500)	500	30	37.8	15.0	39.8

^a^ reaction condition: GL: 2.30 g; Cat: 1.0 g; CO_2_ initial pressure: 4.0 MPa; reaction temperature: 150 °C; reaction time: 5 h; 2-cyanopyridine: 6.32 g; DMF: 19.0 g. ^b^ the number in brackets is the actual CuO content measured by ICP-OES.

**Table 2:** The catalytic activity of CuO/Al_2_O_3_ catalysts with different CuO loading amounts and calcination temperatures for the synthesis of GC from GL and CO_2_ ^a^.

Catalyst	*T*_cal_ (°C)	*W*_CuO_ (wt%) ^b^	*X*_GL_ (%)	*Y*_GC_ (%)	*S*_GC_ (%)
CuO/Al_2_O_3_ (5%, 500)	500	5(4.6)	14.6	2.8	19.2
CuO/Al_2_O_3_ (10%, 500)	500	10(8.2)	17.9	6.0	33.5
CuO/Al_2_O_3_ (20%, 500)	500	20(19.7)	29.4	10.0	34.1
CuO/Al_2_O_3_ (30%, 500)	500	30(23.2)	37.8	15.0	39.8
CuO/Al_2_O_3_ (40%, 500)	500	40(34.4)	51.6	15.3	29.7
CuO/Al_2_O_3_ (30%, 400)	400	30	58.0	15.1	26.0
CuO/Al_2_O_3_ (30%, 600)	600	30	44.0	13.6	30.9
CuO/Al_2_O_3_ (30%, 700)	700	30	41.3	17.5	42.4
CuO/Al_2_O_3_ (30%, 800)	800	30	33.5	14.2	42.4

^a^ Reaction condition: GL: 2.30 g; Cat: 1.0 g; CO_2_ initial pressure: 4.0 MPa; reaction temperature: 150 °C; reaction time: 5 h; 2-cyanopyridine: 6.32 g; DMF: 19.0 g. ^b^ the number in brackets is the actual CuO content measured by ICP-OES.

**Table 3:** The BET surface area, total pore volume, and average pore diameter of CuO/Al_2_O_3_ (30%, 600–800) catalysts.

Catalysts	S_BET_ (m^2^/g)	Total Pore Volume (cm^3^/g)	Average Pore Diameter (nm)	Basic Site Amount (μmol/g)			
				<200 °C	200~400 °C	>400 °C	Total
CuO/Al_2_O_3_ (30%, 600)	111.3	0.269	7.98	171 (0.19) ^a^	367 (0.42)	337 (0.39)	875
CuO/Al_2_O_3_ (30%, 700)	170.5	0.277	6.65	194 (0.18)	185 (0.17)	704 (0.65)	1082
CuO/Al_2_O_3_ (30%, 800)	92.7	0.240	11.42	5 (0.02)	90 (0.27)	234 (0.71)	329

^a^ the number in brackets is the ratio of each basic sites amount to the total basic sites number.

**Table 4:** Comparison of catalytic activity of CuO/Al_2_O_3_ (30%, 700) with the other catalysts for GC synthesis from GL and CO_2_ ^a^.

Catalysts	T (°C)	Time (h)	P (MPa)	Cat. (wt%)	Dehydrant	*X*_GL_ (%)	*Y*_GC_ (%)	STY ^b^	Ref.
CeO_2_	150	5	4	186.8	2-cyanopyride	-	78.9	0.92	[18]
La_2_O_2_CO_3_-ZnO	170	12	4	5	acetonitrile	30.3	14.3	2.59	[31]
CHT-Cl	170	12	4	3.0	acetonitrile	35.5	16.7	4.97	[30]
ZnY	180	3	10	2.0	no	-	5.8	10.58	[24]
Cu/MCM-41	150	3	7	1.7	acetonitrile	18.7	1.8	3.81	[29]
CeO_2_-nanopolyhedra	170	12	10	7.4	2-cyanopyride	35.5	14.2	1.74	[35]
ZnO	180	12	15	0.6	no	-	8.1	11.35	[25]
Co(Oac)_2_	180	6	2	2.5	acetonitrile	36.7	4.6	3.33	[53]
ZnWO_4_-ZnO	150	6	5	54.4	no	-	6.5	0.22	[23]
CuO/Al_2_O_3_(30%, 700)	150	5	4	43.6	2-cyanopyride	41.3	17.5	0.87	This work

^a^ T: reaction temperature; Time: reaction time; P: reaction pressure of CO_2_; Cat.: catalyst amount based on GL weight. ^b^ STY = mol of GC produced/(weight of catalyst reaction time) (mmol/gcat∙h).

## Data Availability

The authors state that the data pertaining to the manuscript will be made available upon request.

## Sample Availability

Samples of the compounds GC are available from the authors.
